# Kurze Geschichte der Katheterablation mit DC-Schocks

**DOI:** 10.1007/s00399-024-01011-3

**Published:** 2024-02-29

**Authors:** Helmut U. Klein, Hans-Joachim Trappe, Günter Frank

**Affiliations:** 1 Hannover, Deutschland; 2 Dülmen, Deutschland; 3 Lübeck-Travemünde, Deutschland

**Keywords:** DC-Katheterablation, Atrioventrikuläre Überleitung, Vorhofflimmern, WPW-Syndrom, Kammertachykardie, Gleichstrom Ablation, DC catheter ablation, Atrioventricular conduction, Atrial fibrillation, Wolff-Parkinson-White syndrome, Ventricular tachycardia, DC Ablation

## Abstract

Ein erster Bericht über eine DC-Katheterablation bei 5 Patienten zur Unterbrechung einer schnellen AV-Überleitung bei Vorhofflimmern und anschließender Pacemaker-Implantation erschien 1982 von Scheinmann et al. (San Francisco, CA, USA). In Deutschland berichteten 1984 Seipel, Breithardt und Borggrefe (Düsseldorf) und 1985 Manz und Lüderitz (Bonn) über erste Erfahrungen mit der DC-Katheterablation. Ein erstes internationales DC-Ablationsregister, an dem auch vier deutsche Zentren beteiligt waren, berichtete 1984 über 127 Patienten aus 24 Zentren. Ein kompletter AV-Block wurde bei 71 % der Patienten erreicht. Die Hannover Gruppe (Trappe, Klein und Huang) berichtete 1992 über Ergebnisse der DC-Katheterablation der AV-Leitung zwischen 1983 und 1990 bei 100 Patienten (86 % Vorhofflimmern, 14 % AV-Reentry Tachykardien). Eine erste erfolgreiche DC-Katheterablation bei einem WPW-Syndrom berichtete Morady (San Francisco, CA) 1985. Borggrefe et al. haben 1987 erstmals über einen Wechsel von der DC-Katheterablation auf ein HF-Katheterverfahren bei einem WPW-Syndrom berichtet. Erste DC-Katheterablationen bei Kammertachykardien beschreibt Hartzler (Kansas City, MO, USA) 1983 bei 3 Patienten. Borggrefe et al. berichten 1989 über 24 Patienten, bei denen eine DC-Katheterablation wegen Kammertachykardien durchgeführt wurde; 17 Patienten hatten nach 14 Monaten keine VT-Rezidive. Die Hannover Gruppe (Trappe, Klein) berichtete 1994 über die Langzeitergebnisse der DC-Katheterablation bei Kammertachykardien nach 5 Jahren bei 51 Patienten. Die VT-Rezidivrate (57 %) und auch die Gesamtmortalität (16 %) waren hoch. Einen Vergleich von DC-Ablation mit der HF-Ablation bei Kammertachykardien berichtete Gonska (Göttingen) 1994. Die Erfolgsraten nach 2 Jahren waren nicht wesentlich unterschiedlich.

## Abstract (en)

Direct current (DC) catheter ablation in 5 patients aiming to interrupt rapid atrioventricular (AV) conduction with atrial fibrillation and subsequent pacemaker implantation was first published by M. M. Scheinman et al. (San Francisco, CA, USA) in 1982. In Germany, L. Seipel, G. Breithardt, and M. Borggrefe reported their first experience with DC catheter ablation in 1984, followed by the group in Bonn (M. Manz and B. Lüderitz) in 1985. The first international DC catheter ablation registry, which also included four German centers, reported DC catheter ablation results of 127 patients in 24 centers in 1984. Complete AV block was achieved in 71% patients. In 1992, the Hannover group (H‑J. Trappe, H. Klein and J. Huang) reported results of DC catheter ablation of AV conduction performed between 1983 and 1990 in 100 patients (86% with rapid atrial fibrillation, 14% with AV-node reentry tachycardias). The first successful DC catheter ablation in a patient with Wolff–Parkinson–White (WPW) syndrome was reported in 1985 by F. Morady et al. (San Francisco, CA, USA). In 1987, M. Borggrefe et al. were the first to report a switch from DC catheter ablation to a high-frequency (HF) catheter ablation procedure in a patient with WPW syndrome. The use of DC catheter ablation to treat ventricular tachycardia (VT) was described by G. O. Hartzler (Kansas City, MO, USA) in 3 patients in 1983. M. Borggrefe et al. (1989) reported on 24 patients who underwent DC catheter ablation for VT. Of those, 17 patients did not have VT recurrence within the following 14 months. In 1994, the Hannover group (H-J Trappe, H. Klein) published their 5‑year long-term results of DC catheter ablation of VT in 51 patients. VT recurrence occurred in 57% patients and overall mortality was also high (16%). A comparison of DC catheter ablation with HF catheter ablation for recurrent VT was reported in 1994 by G. Gonska et al. (Göttingen, Germany). After 2 years follow-up, success rates were not found to be significantly different.

Nur wenige Jahre nach den ersten chirurgischen Behandlungen von Tachykardien wurde bereits 1982 über die ersten Ergebnisse einer Katheterablation mit Gleichstrom (DC) zur Unterbrechung der atrioventrikulären Überleitung mit Hilfe eines mehrpoligen Elektrodenkatheters berichtet. Was zunächst als „elektrischer Unfall“ bei einer elektrophysiologischen Untersuchung durch Kontakt des Elektrodenkatheters mit dem Elektrodenpaddel bei einer Defibrillation und resultierendem AV-Block beschrieben wurde, entwickelte sich schließlich zu einem neuen Therapieverfahren [1]. Das Ziel war, bei Patienten mit medikamentös nicht kontrollierbarer schneller AV-Überleitung bei Vorhofflimmern, Vorhofflattern, AV-Knoten-Reentry-Tachykardien oder damals so bezeichneten *ektopen* atrialen Tachykardien eine Normalisierung der Herzfrequenz durch AV-Leitungsunterbrechung oder wenigstens Verlangsamung der AV-Leitung mit folgender RV-Pacemaker Implantation zu erreichen. Scheinman aus San Francisco berichtete 1982 über 5 Patienten, bei denen nach 3–5 DC-Schocks mit 300–500 J über einen modifizierten Elektrodenkatheter eine komplette AV-Blockierung erreicht wurde [2]. Bei Allgemeinnarkose wurden synchronisierte, monophasische DC-Schocks − manchmal bis zu 6 Schocks − von kommerziellen externen Defibrillatoren zwischen der distalen Katheterelektrode als Anode und einer externen *Patchelektrode* als Kathode abgegeben. Im gleichen Jahr berichtete Gallagher über 9 Patienten mit erfolgreicher Blockierung der AV-Überleitung durch DC-Schocks (200–300 J) über handelsübliche tripolare Elektrodenkatheter [3].

In den folgenden Jahren berichteten auch mehrere Gruppen in Deutschland über ihre Erfahrungen mit der transvenösen DC-Katheterablation. 1984 war es die Düsseldorfer Gruppe mit Seipel, Breithardt und Borggrefe [4] und 1985 die Bonner Gruppe mit Manz und Lüderitz, die über 15 Patienten mit DC-Ablation bei AVNRT, Vorhofflattern und junktionalen Tachykardien berichteten [5]. Da nur bei 10 von 15 Patienten eine komplette Blockierung der AV-Leitung erreicht wurde, wertete Manz die Ergebnisse der „His-Bündel-Ablation“ als mäßig zufriedenstellend. Ergebnisse eines weltweiten, internationalen Registers zur Unterbrechung der AV-Überleitung bei 127 Patienten aus 24 Zentren mit supraventrikulären Tachykardien wurden 1984 von Scheinman berichtet [6]. An diesem Register nahmen auch vier deutsche Zentren teil. Ein kompletter AV-Block nach 1–6 Schocks wurde nur bei 71 % der Patienten erreicht. Bei der Nachbeobachtung verstarben 3 Patienten plötzlich, bei 5 Patienten wurde nach erfolgloser DC-Ablation eine chirurgische Unterbrechung der AV-Leitung durchgeführt. Die Gruppe in Hannover mit Trappe, Klein und Huang berichtete 1992 über ihre Erfahrungen mit der DC-Katheterablation der atrioventrikulären Überleitung zwischen 1983 und 1990 bei 100 Patienten [7]. Ein medikamentös nicht kontrollierbares Vorhofflimmern bestand bei 86 Patienten; bei 14 Patienten bestand eine AV-Knoten-Reentry-Tachykardie. Eine komplette Unterbrechung der AV-Leitung nach der ersten Ablationssitzung (2–8 Schocks mit 360–400 J) wurde bei 85 % der Patienten erreicht. Bei 13 Patienten wurde eine zweite Ablationssitzung durchgeführt. Nach einer mittleren Nachbeobachtungszeit von 44 Monaten waren 13 Patienten verstorben, ein Patient durch einen plötzlichen Herztod. Komplikationen traten bei 18 % der Patienten auf; die gefährlichsten waren die akuten Perikardtamponaden. Von 1983–1985 wurden nur festfrequente VVI-Schrittmacher und seit 1986 dann sog. frequenzadaptierte Schrittmacher direkt nach der Ablation implantiert.

Während die englischsprachigen Publikationen berichten, dass die erste DC-Katheterablation bei einem WPW-Syndrom von Fisher 1984 im Koronarsinus erfolglos durchgeführt wurde [8], und dann 1985 über einige Ablationen von akzessorischen Bündeln im posteroseptalen Bereich von Morady und Scheinman berichtet wurde [9], muss man feststellen, dass bereits 1983 Weber und Schmitz aus Göttingen einen kurzen Beitrag im *New England Journal of Medicine* über einen Fall einer DC-Ablation eines retrograd leitenden akzessorischen Bündels im rechten Vorhof berichtet haben [10]. Die Hamburger Gruppe mit Kunze und Kuck berichtete 1984 in einem *Circulation*-Abstract ebenfalls über eine WPW-Ablation mit Elektroschocks [11]. Borggrefe aus der Düsseldorfer Gruppe berichtete 1987 über einen Fall, bei dem die DC-Katheterablation eines rechtsatrialen akzessorischen Bündels erfolglos blieb, und dann 3 Tage später eine HF-Katheterablation mit dem später allgemein benutzten Osypka HAT 100 Generator mit 2 × 50 W erfolgreich durchgeführt wurde [12]. Das war vielleicht in Deutschland die Einleitung der Hochfrequenz-Katheterablation (HF- oder RF-Ablation; [13, 14]). Die Hannover Gruppe mit Klein, Trappe hat 1986 zusammen mit Kallfelz und Luhmer bei einem 2 Wochen alten, männlichen Neugeborenen mit einer medikamentös nicht zu terminierenden („incessant“) supraventrikulären Tachykardie über einen 5‑F-Elektrodenkatheter, der über die noch vorhandene Umbilikalvene eingeführt wurde, eine komplette Unterbrechung der AV-Überleitung mit 3 DC-Schocks (12 J, 24 J, und 50 J) erreicht. Nach 3 Wochen eines kompletten AV-Blocks mit temporärer Stimulation im RV trat einen Tag vor der geplanten epikardialen Schrittmacherimplantation erstmals spontan ein regelrechter Sinusrhythmus mit normaler AV-Überleitung ein. Bei einer mehr als 20-jährigen Nachbeobachtung ist niemals wieder eine Tachykardie aufgetreten. Wahrscheinlich hat es sich bei dieser DC-Ablation der *AV-Leitung* ungewollt um eine Mahaim-Fiber-Ablation gehandelt. Leider ist dieser Fall niemals publiziert worden. Vielleicht aber war diese DC-Ablation ein Eingriff an einem der jüngsten Patienten, die jemals durchgeführt wurde.

1983, ein Jahr nachdem Scheinman über seine ersten Erfahrungen zur DC-Katheterablation der AV-Überleitung berichtet hatte, erschien die erste Publikation zur DC-Katheterablation bei ventrikulären Tachykardien von Hartzler [15]. Bei einer jungen Patientin erfolgte die Schockabgabe im RV-Ausflusstrakt, bei zwei männlichen Patienten nach Myokardinfarkt und rezidivierenden VT im LV-Septum. Mit diesen ersten DC-Ablationen wegen VT eröffnete sich ein neues Feld, weg von den kompletten Unterbrechungen der AV-Überleitung und den nicht sehr überzeugenden Ergebnissen der Ablation akzessorischer Leitungsbahnen. Dennoch blieb auch hierbei die Zahl der Zentren, die DC-Katheterablationen durchführten und über Behandlungsergebnisse berichteten, gering. Das lag einerseits an der relativ aufwendigen Prozedur mit erforderlicher Anästhesie und andererseits an der nicht geringen Komplikationsrate. Borggrefe et al. berichteten 1989 über die Ergebnisse der DC-Ablation bei 24 Patienten mit Kammertachykardien [16]. Nach ausführlichem Katheter-Mapping wurden 2–6 DC-Schocks mit 100–400 J im Bereich der frühesten Erregung oder der Zone der größten Leitungsverzögerung über die distale Elektrode konventioneller Elektrodenkatheter abgegeben. Ein Patient verstarb während der Prozedur durch eine Ventrikelruptur; innerhalb einer Woche entwickelten 9 Patienten (37 %) ein Tachykardie-Rezidiv. Bei 7 Patienten wurde eine zweite oder dritte Prozedur durchgeführt; insgesamt lag die Komplikationsrate durch oder nach der Ablation inklusive Perikardergüsse bei 30 %. Während der 14-monatigen Nachbeobachtung blieben 17 Patienten (71 %) ohne erneute VT. Die Hannover Gruppe um Trappe und Klein berichtete 1992 über ihre Langzeitergebnisse der DC-Ablation bei anhaltenden VT [17], und dann letztmalig 1994 über ihre Langzeitergebnisse der Katheterablation mit der DC-Technik bei Kammertachykardien bei 51 Patienten [18]. Nach 5 Jahren traten bei 29 Patienten (57 %) VT-Rezidive auf; mehrheitlich bei Patienten mit VT bei nichtischämischer Kardiomyopathie; 8 Patienten (16 %) verstarben innerhalb dieser Zeit, zwei durch plötzlichen Herztod. Interessant war, dass VT-Rezidive mehrheitlich bei solchen Patienten auftraten, bei denen die DC-Energieabgabe am Ort der – durch Mapping ermittelten – frühesten Erregung während der VT erfolgte, aber selten bei DC-Schock-Abgaben in Gebieten mit verzögerter Erregung (fraktionierte Elektrogramme oder Spätpotenziale). Dies lässt vermuten, dass durch die DC-Energie-Abgabe das Milieu des *arrhythmogenen* Areals verändert wurde, jedoch nicht der Ursprungsort der VT ausgeschaltet werden konnte. Fast alle Patienten mit VT-Rezidiven erhielten danach implantierbare Defibrillatoren.

Da die ersten Berichte zur möglichen Anwendung einer Ablation mit Hochfrequenz-Technik (RF-Ablation) schon 1985 erschienen, war es nur eine Frage der Zeit, dass die DC-Ablationstechnik verlassen werden konnte. Eine interessante Arbeit erschien dann noch 1994 von der Göttinger Gruppe um Gonska et al., die über einen Vergleich zwischen DC-Schock-Ablation mit Hochfrequenz-Katheterablation von monomorphen Kammertachykardien bei ausschließlich koronarer Herzkrankheit berichteten [19]. Die Ergebnisse von 72 Patienten mit Hochfrequenzablation der VT wurden mit 64 Patienten verglichen, die eine DC-Katheterablation ihrer Kammertachykardie erhielten. Bei allen Patienten wurde ein endokardiales Katheter-Mapping zur Lokalisation des VT-Ursprungs vor der Ablation durchgeführt. Es zeigte sich, dass die Erfolgsrate bei beiden Ablationsmethoden etwa gleich war (Initialerfolg bei HF-Ablation 74 %, bei DC-Ablation 77 %); bei einer mittleren Nachbeobachtung von 2 Jahren war die VT-Rezidivrate 20 % bei HF-Ablation und 13 % bei DC-Ablation. Bei beiden Ablationsmethoden trat jeweils ein prozedurbedingter Todesfall auf. Wenn durch programmierte Stimulation die VT auslösbar blieb, erhielten die meisten Patienten dann einen implantierbaren Defibrillator. Trotz vergleichbarer Resultate sagten die Autoren voraus, dass die Zukunft der Katheterablation der HF-Ablationstechnik gehören würde.

Die Katheterablation mit der DC-Technik, die von der französischen Arbeitsgruppe um Fontaine als „Fulguration“ bezeichnet wurde [20], blieb eine kurze, weniger als 8 Jahre dauernde Episode der Rhythmologie. Sie wurde schnell von der HF-Ablation abgelöst. Andere Verfahren wie die Kryoablation, verschiedene HF-Techniken und Energie Formen, wie jetzt auch die *High-power-short-duration-Ablation* und die neue Form der *Pulse-field-Ablation* sind heute die Methoden der Katheterablation. Der medizinisch-technische Aufwand der DC-Ablation war groß, benötigte immer eine Vollnarkose, die Erfolge waren nicht überzeugend, und die Komplikationen waren nicht gering. Eine rasante Entwicklung der Mapping-Technologie und der elektromagnetischen dreidimensionalen kardialen Bildgebung haben den schnellen Übergang zur HF-Ablation erheblich erleichtert.
